# A Critical Review of Glucose Biosensors Based on Carbon Nanomaterials: Carbon Nanotubes and Graphene

**DOI:** 10.3390/s120505996

**Published:** 2012-05-10

**Authors:** Zhigang Zhu, Luis Garcia-Gancedo, Andrew J. Flewitt, Huaqing Xie, Francis Moussy, William I. Milne

**Affiliations:** 1 Electrical Engineering Division, Department of Engineering, University of Cambridge, J J Thomson Avenue, Cambridge, CB3 0FA, UK; E-Mails: lg371@cam.ac.uk (L.G.-G.); ajf@eng.cam.ac.uk (A.J.F.); wim1@cam.ac.uk (W.I.M.); 2 School of Urban Development and Environmental Engineering, Shanghai Second Polytechnic University, Shanghai 201209, China; E-Mail: hqxie@eed.sspu.cn; 3 Brunel Institute for Bioengineering, Brunel University, Uxbridge, Middlesex, UB8 3PH, UK; E-Mail: moussyf@who.int; 4 Department of Information Display, Kyung Hee University, 1 Hoegi-dong, Dongdaemun-gu, Seoul 130-701, Korea

**Keywords:** glucose biosensor, carbon nanotube, graphene, non-enzymatic sensor, nanotechnology

## Abstract

There has been an explosion of research into the physical and chemical properties of carbon-based nanomaterials, since the discovery of carbon nanotubes (CNTs) by Iijima in 1991. Carbon nanomaterials offer unique advantages in several areas, like high surface-volume ratio, high electrical conductivity, chemical stability and strong mechanical strength, and are thus frequently being incorporated into sensing elements. Carbon nanomaterial-based sensors generally have higher sensitivities and a lower detection limit than conventional ones. In this review, a brief history of glucose biosensors is firstly presented. The carbon nanotube and grapheme-based biosensors, are introduced in Sections 3 and 4, respectively, which cover synthesis methods, up-to-date sensing approaches and nonenzymatic hybrid sensors. Finally, we briefly outline the current status and future direction for carbon nanomaterials to be used in the sensing area.

## 1. Introduction

Diabetes is a group of metabolic diseases affecting about 150 million people worldwide, and is one of the leading causes of death and disability, such as blindness, nerve degeneration and kidney failure [1–3]. The diagnosis and management of diabetic patients require precise monitoring and control of the glucose level in the body. Therefore, frequent testing of the physiological glucose level is critical to confirm treatment efficiency, prevent long-term complications and avoid a diabetic emergency, such as hypoglycaemia (low blood sugar, <3 mM). Currently, diabetics must frequently check their blood glucose levels by “finger-pricking” and adjust their insulin dosage to keep the glucose level as close to “normal” as possible. Tens of millions of glucose assays have been used for diabetic tests, and glucose biosensors thus account for about 85% of the entire sensing market. So far, three generations have been developed for this huge market and the global market for glucose biosensors and strips will reach $11.5 billion by 2012, according to the recent report by Global Industry Analysts, Inc. Regular finger-pricking tests neglect night time variations and may result in poor approximation of blood glucose variations. Therefore, continuous monitoring is agreed to be the best way to solve this problem, and is being considered for next generation products to replace the currently used household glucose meters. Before a glucose sensor can provide continuous measurement, it must be placed in the body, which often leads to difficulties, such as biofouling, fibrous capsule, inflammation, loss of vasculature and loss of function [4]. We will not focus on implantable sensors; review papers on these can be found elsewhere [5–8]. However, some concepts regarding the usage of carbon nanomaterials, such as CNT fiber, in implantable sensors will be briefly mentioned in this paper.

Sensors continue to have a significant impact in everyday life. There has been a strong demand to produce highly selective, sensitive, responsive, and cost effective sensors. Carbon might be the most widely-used material in electroanalysis and electrocatalysis for sensing. Carbon-based nanomaterials, especially CNTs and graphene, are extremely attractive in the bioanalytical area for electrode design as they can combine properties of the high surface area, acceptable biocompatibility, chemical and electrochemical stability and good electrical conductivity [9–11]. The conductivity, along with the small size of carbon nanotubes, make them suitable as individual nanoelectrodes, and many studies have shown that the electric properties of these individual nanoelectrodes have the ability to efficiently promote electron-transfer reactions [12,13]. The integration of CNT into biosensing electrodes has been challenging. Often, they have been used as intermediates between platinum, gold or glassy carbon electrodes and enzymes. Random dispersions of CNTs on Pt, Au or glassy carbon electrodes were achieved by using a binder to form a CNT paste [14], or by making composites with other materials including Teflon and polyvinyl acetate (PVA) [15]. To gain greater control over the distribution of nanotubes, Wohlstadter *et al.* were able to control the orientation of nanotubes using polymer extrusion [16]. Liu *et al.* provided a more versatile approach for organizing randomly tangled single walled carbon nanotubes (SWCNTs) on gold surfaces by self-assembly using spontaneous chemical binding via Au-S bonds [17]. To overcome the lack of robustness of self-assembled nanotubes, Lin *et al.* developed amperometric biosensors based on CNT-nanoelectrode ensembles (NEEs), where millions of nanoelectrodes were embedded in epoxy, and the enzyme immobilized on the NEEs through carbodiimide chemistry [18].

Graphene ideally forms two dimensional structures and comprises a single layer of sp^2^-hybridized carbon atoms joined by covalent bonds to form a flat hexagonal lattice. An isolated single layer of graphene is difficult to fabricate [19]. Although the electrochemical properties of graphene are not clearly understood, most work has confirmed that fast electron transfer between enzymes and electrodes can be obtained due to the unique electronic structure of graphene [20–22]. This mainly comes from the delocalized π bonds above and below the basal plane. These delocalized electrons create high electrical conductivities and mobilities for graphene within the plane [23]. The use of graphene in biosensors can avoid the problem associated with the transition metals, like Fe, Ni, Co, *etc.* The fast electron transportation, high thermal conductivity, excellent mechanical flexibility and good biocompatibility make the graphene an ideal candidate for biosensing application.

This review particularly focuses on the nanomaterials, carbon nanotubes and graphene, in the field of glucose biosensors. A brief history of three generations of glucose biosensors is firstly presented in Section 2. The synthesis of carbon nanotube, CNT pasted electrode, field-effect transistor, CNT fiber and non-enzymatic based sensors are introduced in Section 3. The synthesis of graphene, graphene based enzymatic and non-enzymatic sensors are mentioned in Section 4. Finally, we briefly outline the current status and future direction for carbon nanomaterial which are being targeted toward the glucose sensing area.

## 2. Brief History of Glucose Biosensors

The first glucose sensor was developed by Clark and Lyons from the Cincinnati Children's Hospital [24], and the electrode relied on a layer of glucose oxidase (GOD) entrapped over an oxygen electrode through the following reaction:

(1) $$\text{Glucose}+{\text{O}}_{2}\;\overset{\text{GOD}}{\to }\;\text{Gluconic acid}+{\text{H}}_{2}\text{O}$$

then, a negative potential was applied to the cathode to detect oxygen consumption:

(2) $${\text{O}}_{2}+4{\text{H}}^{+}+4{\text{e}}^{-}\to 2{\text{H}}_{2}\text{O}$$

The first product based on the above technology was commercialized by the Yellow Spring Instrument company (YSI) using only 25 μL whole blood samples. The entire glucose sensor market has grown rapidly since then.

### 2.1. First-Generation Sensors

The first-generation glucose biosensors rely on the use of yellow glucose enzyme, which involves the use of oxygen and thus the generation and detection of hydrogen peroxide. The reduction of the flavin adenine dincleotide (FAD) in the enzyme with glucose results in the reduced form of the enzyme FADH_2_:

(3) $$\text{GOD}(\text{FAD})+\text{glucose}\to \text{GOD}({\text{FADH}}_{2})+\text{gluconolactone}$$

The reoxidation of the flavin with free oxygen generates the oxidized form of the enzyme FAD:

(4) $$\text{GOD}({\text{FADH}}_{2})+{\text{O}}_{2}\to \text{GOD}(\text{FAD})+{\text{H}}_{2}{\text{O}}_{2}$$

The measurements of the hydrogen peroxide lead to simple design of the devices and it helps to miniaturize the sensors. A typical product that entrapped the GOD between the inner anti-interference cellulose acetate membrane and outer diffusion-limiting membrane was firstly invented by the YSI company.

One issue concerning the first generation is electroactive interference, since a relatively high potential was needed to measure the H_2_O_2_. This high potential leads to endogenous reducing species, such as ascorbic and uric acids and some drugs, like acetaminophen [25]. Two main efforts have been applied to minimize the interference effect: (i) use of a selective membrane to minimize the interferents toward the electrode, for example by coating with a Nafion and cellulose acetate layer; (ii) reducing the operative potentials to an optimal region (0–0.2 V *vs.* Ag/AgCl) to avoid the electroactivity of the interferents.

Another issue concerning the first generation is the oxygen dependence. As shown in Equation (4), the oxygen amount is a limiting factor (oxygen deficit) that controls the changes in sensor response and the upper limit of linearity. Several efforts have been suggested to address this problem: (i) mass-transport limiting films, such as polyurethane or polycarbonate, were selected to tailor the flux of glucose and oxygen [26,27]; (ii) another approach involves oxygen-rich carbon paste enzyme electrodes, which have high oxygen solubility and can act as an internal source of oxygen [28].

### 2.2. Second-Generation Sensors

Glucose oxidase does not directly transfer electrons to traditional electrodes because the FAD redox center is surrounded by a thick protein layer and this blocks the direct electron transfer. Therefore, employing a nonphysiological electron acceptor to shuttle electrons and solve the oxygen deficiency is the main approach in this generation of sensors.

**Figure 1.**
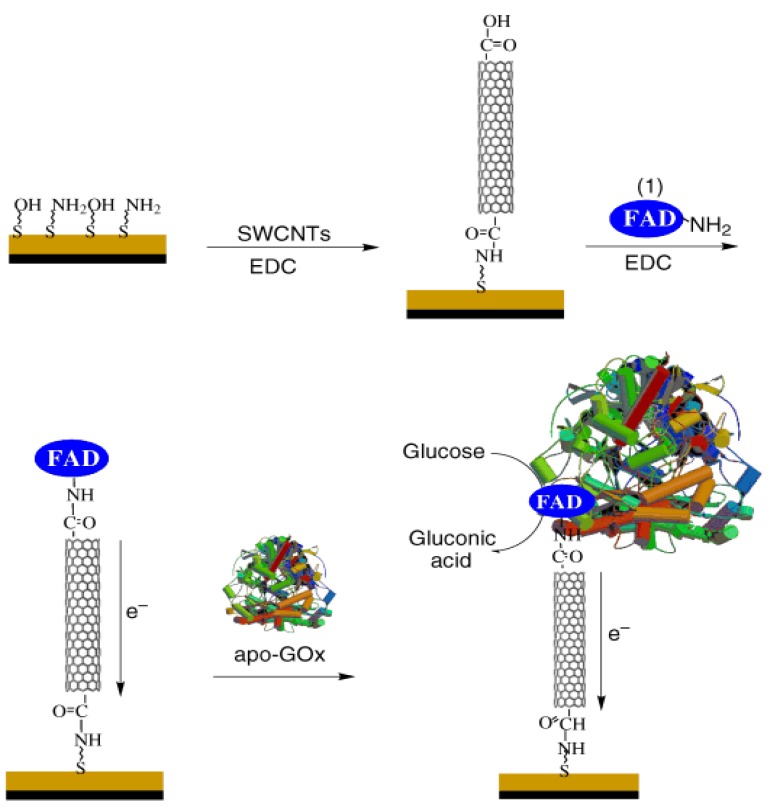
Assembly of the SWCNT electrically contacted glucose oxidase electrode. A 2-thioethanol/ cystamine mixed monolayer (3:1 ratio) was assembled on an Au electrode and the length fractionalized SWCNTs were coupled to the surface in the presence of the coupling reagent 1-ethyl-3-(3-dimethylaminopropyl) carbodiimide hydrochloride (EDC). The amino derivative of the FAD cofactor (1), was then coupled to the carboxy groups at the free edges of the standing SWCNTs. Finally, Apo-glucose oxidase, apo-GOx, was then reconstituted on the FAD units linked to the ends of the standing SWCNTs. Reprinted with permission from [30].

Redox polymers, like poly(vinylpyridine) or poly(vinylimidazole) covalently linked with osmium-complex electron relays, were able to reduce the distance between the redox center of the polymers and the FAD center of the enzymes, which leads to a high current output and fast sensing response [29]. Nanomaterials, like gold nanoparticles (NPs) or carbon nanotubes, have also been used as electrical connectors between the electrode and the FAD center due to their similar size. For example, Patolsky *et al.* tried to align the enzyme on the electrodes by using SWCNTs as the electrical connectors between the enzyme redox centers and the electrodes [30], FAD was first covalently attached to the SWCNT ends and then GOD was reconstituted at the immobilized FAD, as shown in Figure 1. The results showed the electrode surface was linked to the aligned reconstitution of a redox flavoenzyme on the edge of the carbon nanotubes, and the SWCNTs acted as nanoconnectors that electrically contact the active site of the enzyme and the electrode. The electrons were transported along distances greater than 150 nm and the rate of electron transport is controlled by the length of the SWCNTs.

Nonphysiological electron acceptors, artificial mediators, which shuttle electrons between the FAD center and the electrode surface, are particularly useful and heavily used in the industry to form glucose assays. The reaction can be described as follows [25]:

(5) $${\text{Glucose}+\text{GOD}}_{\left(\text{ox}\right)}\to \text{gluconic acid}+{\text{GOD}}_{\left(\text{red}\right)}$$

(6) $${\text{GOD}}_{\left(\text{red}\right)}+2{\text{M}}_{\left(\text{ox}\right)}\to {\text{GOD}}_{\left(\text{ox}\right)}+2\;{\text{M}}_{\left(\text{red}\right)}+2\;{\text{H}}^{+}$$

(7) $$2{\text{M}}_{\left(\text{red}\right)}\to 2{\text{M}}_{\left(\text{ox}\right)}+2{\text{e}}^{-}$$

where M_(ox)_ and M_(red)_ are the oxidized and reduced forms of the mediator. The reduced form is reoxidized at the electrode, giving a current signal which is proportional to the glucose concentration. Artificial mediators, like ferrocene derivatives, ferricyanide, transition-metal complexes, *etc.* are of particular interest [31,32]. For a good electron-carrying mediator, several critical requirements are necessary: it must (i) react rapidly with the reduced enzyme to minimize competition with oxygen; (ii) possess good electrochemical properties, like low operational potential; (iii) possess low solubility in aqueous medium; (iv) be nontoxic and chemically stable in both reduced and oxidized forms [25].

### 2.3. Third-Generation Sensors

The next generation of glucose sensors tries to eliminate leachable artificial mediators and even the glucose enzyme. It would be a great advance if complicated mediators could be avoided, because elaborate and complicated methods have to be worked out to tether the mediators to the electrodes and enzyme surface. So far, the biggest difficulty is still efficient direct electron transfer between the electrodes and glucose enzymes due to the thick protein around redox center. One possible way to produce third generation glucose sensors containing conducting organic salt electrodes based on charge transfer complexes, such as tetrathiafulvalene-tetracyanoquinodimethane (TTF-TCNQ) [33,34]. Furthermore, mesoporous electrode materials with increased electrode surface and dynamics are gaining more interest [35,36], where direct electron transfer from enzyme to electrode can occur without the complications of mediators and the deficiency of oxygen.

It is believed that the use of non-enzymatic electrodes is another approach for the third generation glucose biosensors. Non-enzymatic glucose electrodes directly oxidize glucose with great sensitivity, the responding current can reach mA·mM^−1^·cm^−2^, and in the meantime, the fragile and expensive glucose enzymes can be avoided. More details about recent progress in the area can be found in the Section 3.6.

## 3. Carbon Nanotube Based Biosensors

CNTs demonstrate faster response time and higher sensitivity than traditional electrodes. The better performance is attributed to their one-dimensional hollow tubular nano-chemistry that is responsible for the efficient capture and promotion of electron transfer from analytes. Furthermore, the dramatic decrease in the overpotentials of hydrogen peroxide observed at CNT-modified electrodes show great promise for application in the glucose sensing area.

### 3.1. CNT Synthesis

Three main methods are used to synthesize SWCNTs and multi-walled carbon nanotubes (MWCNTs): arc-discharge, laser-ablation and chemical vapour deposition (CVD), and each method will be briefly reviewed here.

Arc-discharge is the easiest and most common way to produce CNTs [37]. Indeed, CNTs were firstly discovered by the Japanese scientist, Iijima, who was planning to utilize an arc-discharge method to synthesize fullerenes [38]. The chamber consists of one carbon stick at the cathode and the other at the anode, and can create a plasma by passing 100 A current through the electrode. MWCNTs synthesis by the arc discharge technique is straightforward if two graphite electrodes are introduced, however, a great amount of side products, like fullerenes, amorphous carbon and graphite sheets, are simultaneously formed, which cause difficulty and increase cost as post-deposition purification is required.

Direct laser vaporization of transition-metal/graphite composite rods produced SWCNTs was first introduced by Guo *et al.* [39]. A pulsed or continuous laser beam was introduced into a 1,200 °C furnace to vaporize a target, which is made of graphite and metal catalysts (cobalt or nickel). The merit of the laser-ablation method can be summarized as following: (i) relatively high purity SWCNTs can be synthesized; (ii) a lower temperature furnace can be used with a CO_2_ infrared laser system; and (iii) the quality of CNTs is tuneable by adjusting the nature of the gas and its pressure [40].

CVD synthesis is a better technique for high yield and high purity production of CNT arrays at moderate temperature. The CVD method was firstly used by Yacaman *et al.* [41] and Ivanov *et al.* [42] to produce MWCNTs, and involves the decomposition of gaseous carbon sources. Carbon has a low solubility in these metals at high temperature and CNTs with excellent alignment can be grown perpendicular to the substrate. There are two main types of CVD: thermal CVD and plasma enhanced CVD (PECVD). The carbon nanotubes growth by PECVD have also been studied using some new technologies, like hot filament assisted PECVD [43,44], microwave PECVD [45], dc glow discharge PECVD [46], inductively coupled plasma PECVD [47] and rf PECVD [48]. In thermal CVD, there is essentially a two-step process consisting of a catalyst preparation step followed by the actual synthesis of the nanotube. A higher CVD temperature has positive effects on the crystallinity of the structures produced and can also result in higher growth rates. In some applications, well aligned carbon nanotubes on substrates are desired for this thermal CVD which is uniquely superior to the other methods described above [49–51].

There are great concerns about the impurities in SWCNTs synthesized by the above methods. These impurities are typically removed by acid treatments. However, these acid treatments in turn can introduce other types of impurities, which degrade the nanotube length and perfection, and also increase nanotube costs. The mixture of semi-conducting and metallic tubes in the grown SWCNTs is another concern when trying to make electronic devices.

### 3.2. CNT Paste Electrode

Wang *et al.* found that increasing the Nafion content (from 0.1 to 5 wt%) resulted in dramatic enhancement of the solubility of both types of CNTs, and thus the use of Nafion as a solubilising agent for CNTs overcomes a major obstacle for creating CNT-based biosensing devices [52]. The CNT/Nafion-coated electrode offered a marked decrease in the overvoltage for the hydrogen peroxide reaction to allow convenient low-potential amperometric detection (−0.05 V *vs.* Ag/AgCl). More experiments based on this work have shown palladium nanoparticles on a Nafion-solubilised CNT film retained their biocatalytic activity and offered an efficient oxidation and reduction of the enzymatically liberated H_2_O_2_, allowing for fast and sensitive glucose quantification. The combination of Pd-GOD electrodeposition with Nafion-solubilised CNTs enhanced the storage time and performance of the sensors. An extra Nafion coating was also used to eliminate common interferents, such as uric and ascorbic acids. The fabricated Pd-GOD-Nafion CNT glucose biosensors exhibited a linear response up to 12 mM glucose and a detection limit of 0.15 mM (S/N = 3) [53].

### 3.3. CNT Array/Forest

**Figure 2.**
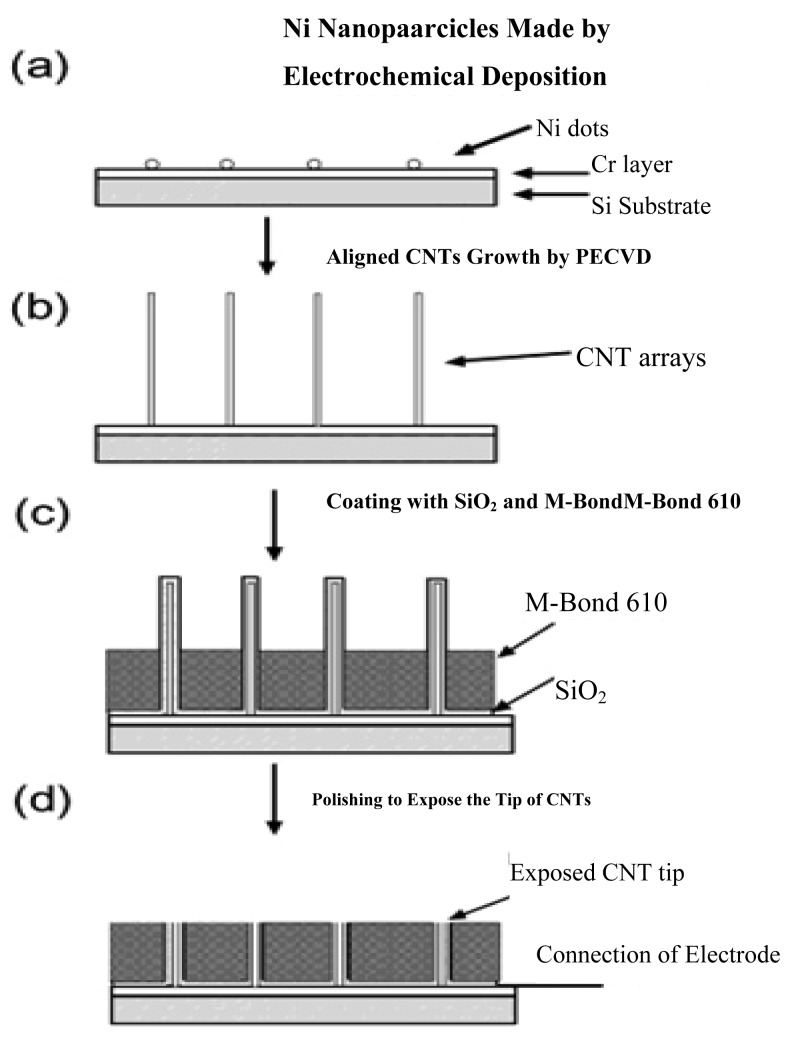
Fabrication scheme of the NEAs. (**a**) Ni nanoparticles electrodeposition; (**b**) aligned carbon nanotube growth; (**c**) coating of SiO_2_ and M-Bond; and (**d**) polishing to expose CNTs. Reprinted with permission from [55].

With the ability of promoting redox reactions of hydrogen peroxide and nicotinamide adenine dinucleotide (NADH), the fabrication of vertically aligned CNTs is an effective way to produce a molecular wire and allow electrical communication between the underlying electrode and a redox enzyme, and thus could be used in amperometric biosensors associated with oxidase and dehydrogenase enzymes [54]. For example, direct electron transfer between FAD of an enzyme and a CNT electrode avoids the requirement of mediators and is thus attractive for the development of reagentless biosensors. Another benefit for the nanotube electrode arrays is their high signal-to-noise ratio and low detection limits (in ppb), owing to the size reduction of each individual electrode and the increased total number of the electrodes. The schematic diagrams for the fabrication of CNT nanoelectrode arrays (NEAs) are depicted in Figure 2.

**Figure 3.**
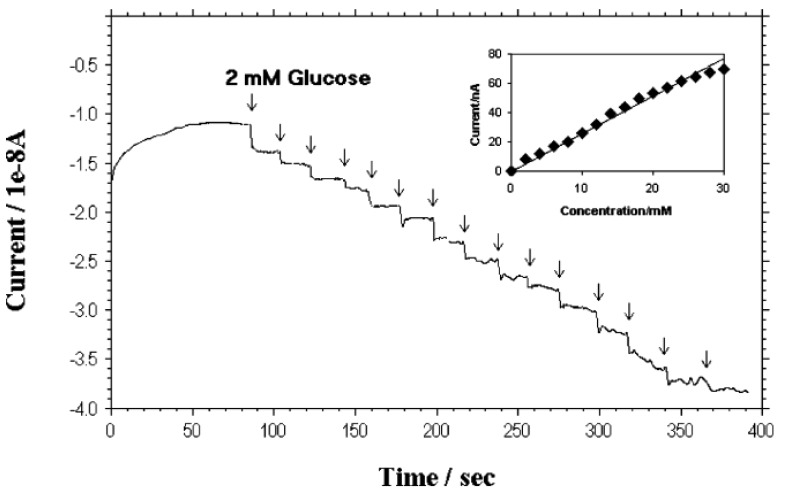
Amperometric responses of a NEE biosensor to successive additions of 2 mM glucose. Reprinted with permission from [18].

Lin *et al.* [18,55] at Pacific Northwest National Laboratory have developed a PECVD method that allows the fabrication of low-site density-aligned carbon nanotubes with an interspacing of more than several micrometers. From this low-site density CNTs, NEAs consisted of millions of nanoelectrodes per cm^2^ where each electrode is less than 100 nm in diameter. They developed these NEAs into mediator-free and membrane-free glucose biosensors. Glucose oxidase was covalently immobilized on CNT NEAs via carbodiimide chemistry by forming amide linkages between their amine residues and carboxylic acid groups on the CNT tips. A linear amperometric response was achieved over physiological levels from 2–30 mM, as shown in Figure 3. The signal response curve is effective at low detection limits at an attractive low-potential point, −0.2 V. The limit of detection can be as low as 0.08 mM based on a signal-to-noise ratio of 3.

A facile strategy has been developed to prepare carbon nanotubes loaded Pt nanoparticle (Pt-CNT) composites. The method involves the polymerization reaction of glucose and the reduction deposition of a platinum source in the pores of anodic alumina membranes (AAMs) under hydrothermal conditions. SEM and TEM images showed that the Pt nanoparticles are uniformly entrapped into the CNTs with a stable hierarchical structure. The nanocomposites electrode is successfully used as a sensitively amperometric sensor for low-potential determination of H_2_O_2_. The as-prepared Pt-CNT-based glucose biosensor displayed a wide linear calibration range of glucose concentrations (0.16−11.5 mM) and a low detection limit of 0.055 mM. Furthermore, the biosensor exhibits some other excellent characteristics, such as high sensitivity and selectivity, short response time, and long-term stability [56]. The stability results show that the electrode remained about 94% of the original value over the first 20 days, and it decreased to 90% after 1 month (use more than 100 times).

### 3.4. CNT FET

**Figure 4.**
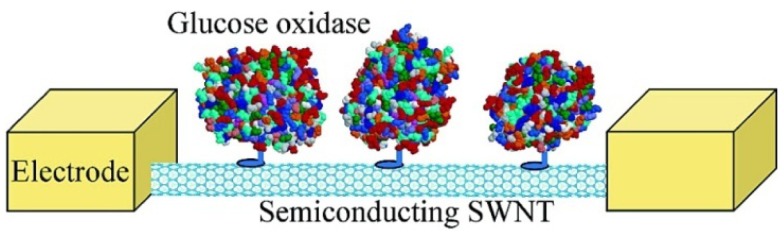
Schematic picture of two electrodes connecting a semiconducting SWCNT with GOD enzymes immobilized on its surface. Reprinted with permission from [57].

There are two advantages of field effect transistor (FET) based biosensors over traditional electrochemical biosensors. FETs detect the electrical signal when the resistance changes due to the absorption of molecules on the FETs surface. Also, they can provide microscale and even nanoscale devices, which can measure the enzymatic activity at the molecular level and are suitable for integration with small chips [23]. Besteman *et al.* firstly introduced SWCNTs into FET biosensing [57]. Controlled attachment of GOD to the nanotube sidewall was achieved through a linking molecule, which on one side bonded to the SWCNT through van der Waals coupling with a pyrene group and on the other side covalently bonded the enzyme through an amide bond, as depicted in Figure 4. The redox enzymes went through a catalytic reaction cycle, where groups in the enzyme temporarily change their charge state and conformational changes occur in the enzyme, which could be detected by the NTFET devices. A step-like response can be monitored in real time after immobilization of GOD in NTFETs.

Li *et al.* recently reported a potentiometric glucose biosensor based on a gate field effect transistor (EGFET) [58]. The biosensor was constructed using a SnO_2_ sensing thin film on an indium tin oxide/polyethyleneterephthalate substrate, chitosan and a mediator of MWCNTs was used. Meanwhile, GOD was entrapped by 3-glycidoxypropyltrimethoxysilane (3-GPTS) using a one-step simple fabrication. The output voltage responses of the potentiometric glucose biosensor were up from 150 mV to 200 mV, and the detection linear range was from 100 mg/dL to 300 mg/dL (1 mM = 18 mg/dL). The biosensor had a 4-day life time and 25-times operational stability.

Lee *et al.* introduced low-cost, transparent, and flexible ion-sensitive field-effect transistors (ISFETs) for glucose sensors [59]. SWCNTs and poly(diallyldimethylammonium chloride, PDDA) are deposited layer-by-layer (LbL) by self-assembly between two metallic electrodes, and was patterned on a polyethylene terephthalate substrate. Glucose is detected by the local pH change in the vicinity of SWCNTs with the aid of GOD enzyme. The glucose sensor shows a sensitivity of 18–45 μA/mM on a linear range of 2–10 mM. The apparent Michaelis-Menten constant is 14.2 mM, indicating a high affinity of LbL assembled GOD to glucose. The LbL self-assembly of nanomaterials and enzymes on the transparent and flexible substrate suggests they are suitable for *in vivo* application.

### 3.5. CNT Fiber

CNT fibers inherit the advantages of high surface area and good electrocatalytic properties of the carbon nanotubes, whilst avoiding potential toxicity caused by asbestos-like CNTs when implanted *in vivo*. Limited efforts have been made to utilize CNT fibers for electrochemical sensors [60–63], and all these studies have used CNT fibers made by a simple particle-coagulation spinning (PCS) process [60]. For example, Wang *et al.* first introduced wet-spun CNT fibers as microelectrodes for electrochemical sensors and demonstrated the possibility of detection of NADH, hydrogen peroxide and dopamine in 2003 [61]. The heat treated MWCNT fiber electrodes respond to NADH over most of the potential range, with significant oxidation currents starting at +0.1 V and levelling off above +0.4 V, which reflects the marked acceleration of the NADH redox process. A CNT fiber microelectrode with proper mediators (2,4,7-trinitro-9-fluorenone) on the surface and various pre-treatments were reported by Viry *et al.* to assemble a biosensor [62,63]. A glucose sensing electrode was built by adsorption of a mediator on the surface of a CNT fiber microelectrode. Electrocatalytic oxidation of analytes via a dehydrogenase works efficiently at 0 V, which is a key point in developing such bioanalytical tools.

**Figure 5.**
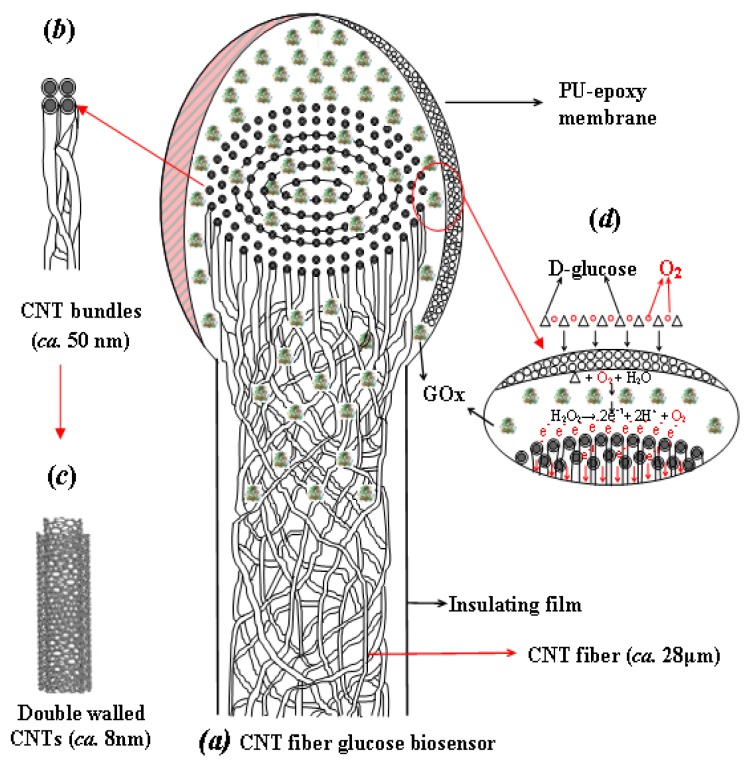
Schematic diagram showing (**a**) CNT fiber based glucose biosensor, (**b**) CNT bundles, (**c**) DWNT and (**d**) working principle of biosensor. The CNT fiber (*ca.* 28 μm) is made of bundles (*ca.* ∼50 nm) of DWNTs (*ca.* 8–10 nm) entangled to form concentrically compacted multiple layers of nano-yarns along the CNT fiber axis, as illustrated in (a). GOx (GOD) enzyme is immobilized at the brush-like end of the CNT fiber and the enzyme layer is encapsulated by the epoxy-polyurethane (EPU) semi-permeable membrane [68].

Recently, a wide variety of continuous yarns of CNTs were fabricated by direct spinning of pure CNT fibers from an aerogel formed during a CVD process using ethanol and acetone as the carbon source [64–67]. The resulting CNT micro-fiber has the potential to address the electrode design and toxicity concerns of CNT based sensors for long-term implantable biosensor applications. During the CVD process, the CNTs formed and self-assembled in the gas flow by van der Waals interactions at high temperature, and then were spun into nano-yarns along the fiber axis. Thus the direct spinning of the pure CNT fiber by the CVD method helped to achieve the best properties in terms of strength, stiffness, toughness, as well as electrical and thermal conductivities, in comparison with the CNT fibers spun from other methods. A comprehensive study about CVD-synthesized CNT fibers used as sensing electrodes to detect glucose solution was introduced by Zhu *et al.* [68]. The specific fiber used was composed of double-walled CNTs that are compacted into concentric layers of CNT bundles organized as nano-yarns [67–69], as shown in Figure 5. The CNT fiber resembles an electric wire, relying on nano-scale surface topography and porosity, which can facilitate molecular-scale interactions with agents like enzymes to efficiently capture and promote electron transfer reactions.

To realize the full potential of the CNT fiber as a biosensor, it was essential to unwind the CNT bundles (nano-yarns) at the ends of the fibers. The resulting brush-like nano-structure resembles a scaled-down electrical “flex” and the individual nano-yarns within the brush-like end would act as multi-nano-electrodes, reminiscent of a dendrite-type nerve cell, which provides many nano-channels to promote and speed up the electron transfer as well as increase the surface area for enzyme immobilization. The whole process of assembling a CNT fiber based biosensor is depicted as Figure 5.

**Figure 6.**
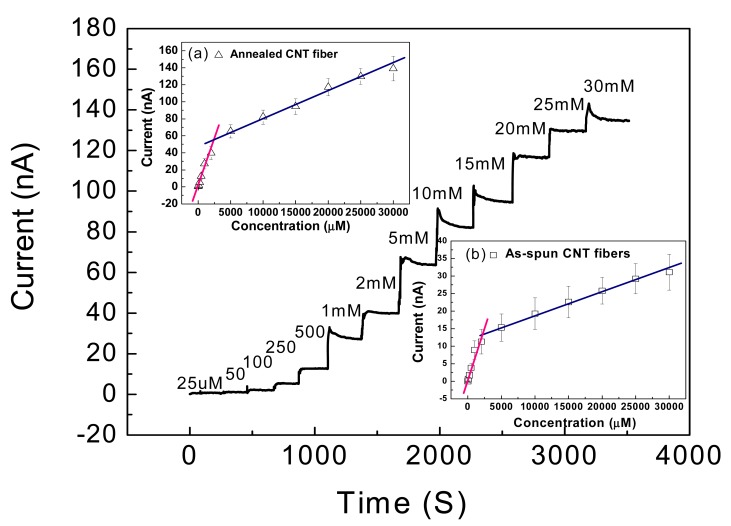
Amperometric responses of CNT fiber biosensors with 30 nm gold film coating. Numbers in the chart represent the corresponding glucose concentration of the solution. The insert shows the calibration plot for annealed CNT fiber (**a**) and as spun CNT fiber (**b**): the linear ranges both divided into two parts: 25 μM to 2 mM and 2 mM to 30 mM [69].

Figure 6 shows the typical amperometric response of the CNT fiber-based glucose biosensor. Fast response of the biosensor towards glucose can be seen in that the sensor response current reaches dynamic equilibrium within tens of seconds (response time) of each addition of glucose, generating a near steady-state current signal. To improve surface conductivity, the CNT fiber was coated with a thin gold film (30 nm) and then connected with Cu wire. As a result, the range of the steady-state current in response to the glucose concentration shifted down to 25 μM and covers the whole range from 25 μM to 30 mM, as shown in Figure 6. This result is much wider than that of the carbon nanofiber based sensor reported by Vamvakaki *et al.* [70]. The detection ranges for both the annealed and as-spun CNT fiber (insert in Figure 6) are divided into two linear sections: 25 μM to 2 mM and 2 mM to 30 mM. The results not only indicate this design should work for traditional diabetic diagnostics due to the linear coverage between 2 mM to 30 mM, but also offer an option of utilization into the more sensitive field. The limit of detection (LOD) of the fiber based sensor is 25 μM, which is the same value as described by zinc oxide nanocomb-based biosensor [71]. The long term stability test showed that the sensitivity remained nearly constant from 10 to 70 days and thereafter a gradual reduction in sensitivity was observed until the end of the study period of 90 days.

### 3.6. Non-Enzymatic Sensors

Most glucose biosensors are based on a GOD because the GOD is able to identify glucose target molecules quickly and accurately through catalyzing glucose to gluconic acid and H_2_O_2_ [68]. However, common and serious problems about the GOD are the price and insufficient long-term stability. It can be easily affected by temperature, pH value, humidity and toxic chemicals, due to the nature of enzymes [72]. Beyond that, complicated procedures (including adsorption, cross-linking, entrapment and electropolymerization) are required to immobilize enzymes on solid electrodes, leading to a decrease in the activity of the enzymes [72,73]. Metal/metal oxide based catalysts, like Cu [73], CuO [74], MnO_2_ [75] and Ni [76] were applied to modify the electrodes for non-enzymatic determination of glucose. Electrooxidation of glucose to glucolactone can be attributed to the redox reaction of metal, like M(III)/M(II) (M = Ni or Cu), based on the following steps:

(8) $$\text{β‐NiOOH}+\text{glucose}\to \text{β‐Ni}{\left(\text{OH}\right)}_{2}+\text{glucolactone}$$

or:

(9) $$\text{γ‐NiOOH}+\text{glucose}\to \text{α‐Ni}{\left(\text{OH}\right)}_{2}+\text{glucolactone}$$

To further improve the sensitivity, carbon nanotubes can be employed because the CNTs are able to promote fast electron transfer kinetics for glucose oxidation [77] and provide large surface to volume ratio [78].

#### 3.6.1. Nickel Modified CNT Array

We have reported a facile yet efficient route for the preparation of well-dispersed Ni nanoparticles on vertically aligned CNTs grown directly on a Si/SiO_2_ substrate [79]. One benefit of this approach is that the density of CNTs forest (10^9^ cm^−2^) is significantly lower than that used in previous reports (10^11^–10^12^ cm^−2^) [80,81], which enables the sputtered Ni nanoparticles to be deposited inside and on top of the CNT forest rather than only aggregating on the top of CNTs as in the dense CNT arrays.

**Figure 7.**
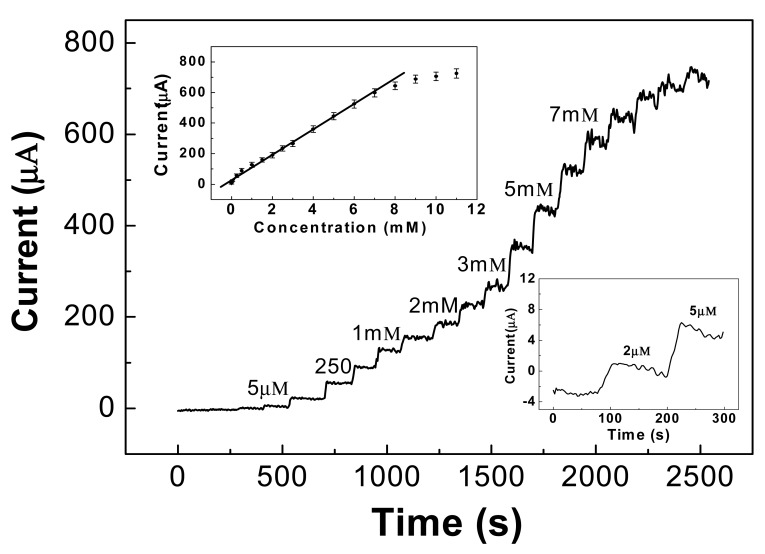
Amperometric response of CNT/Ni electrodes at 0.55 V upon addition of glucoses in 0.1 M NaOH solution. Upper-inset: The calibration curve of current *vs.* concentration of glucose; Error bars indicate the standard deviations of three measurements. Lower-inset: Amperometric response to 2 and 5 μM glucose [79].

A typical amperometric response curve for glucose at various concentrations in a 0.1 M alkaline solution is illustrated in Figure 7, at a +0.55 V applied potential. The sensitivity of the CNT/Ni electrode can reach as high as 1,433 μA·mM^−1^·cm^−2^, received from the slope of the linear regression equation divided by the working surface area, which is much higher than most nonenzymatic glucose sensors reported so far, such as those based upon Cu/MWCNTs (251.4 μA·mM^−1^·cm^−2^) [73]. The calibration plot for glucose determination was linear over a wide range between 5 μM and 7 mM, as shown in the upper-inset of Figure 7. The regression equation is *I_pa_* (μA) = 16.48 + 85.96 c (mM), with a correlation coefficient of 0.9902. The detection limit is able to reach 2 μM, based on the signal/noise ratio of 3, as shown in the lower-inset of Figure 7.

#### 3.6.2. Copper Modified CNT Array

Copper nanoparticles can be considered in the same way as the Ni electrode to catalyse glucose oxidation by a redox couple Cu(III)/(II). Yang *et al.* [82] successfully fabricated vertically well-aligned MWCNTs array and electrochemically deposited CuO nanoparticles onto the sidewalls and tips of MWCNTs by a two-step electrodeposition method. The CuO-modified MWCNTs displayed substantially higher electrocatalytic activity to glucose oxidation with a higher current response and lower oxidation potential than the unmodified MWCNTs. This CuO–MWCNT electrochemical sensor has a low detection limit of 800 nM and a very high sensitivity of 2,190 μA·mM^−1^·cm^−2^, and the response is linear up to 3.0 mM glucose concentration. When these superior performance characteristics are combined with ease of fabrication, long-term stability, good reproducibility, rapid response, and excellent specificity to glucose in the presence of common interferents, the CuO-MWCNTs electrode is a potential candidate for routine glucose analysis.

Another approach showed that the CuO/MWCNTs electrode presented a high sensitivity of 2,596 μA·mM^−1^·cm^−2^ to glucose [74], at an applied potential of +0.40 V. In addition, the linear range was obtained over a concentration up to 1.2 mM with a detection limit of 0.2 μM (signal/noise = 3). The response time is about 1 s with addition of 0.10 mM glucose. More importantly, the CuO/MWCNTs electrode is also highly resistant against poisoning by chloride ions, and the interference from the oxidation of common interfering species such as ascorbic acid, dopamine, uric acid and carbohydrate compounds is effectively avoided. In addition, the CuO/MWCNTs electrode was also used to analyze glucose concentration in human serum samples. The sensitivity, linear calibration range and detection limit are listed in Table 1 to compare the CNT based electrode with other non-enzymatic sensors reported recently [79].

## 4. Graphene Based Biosensors

The discovery of graphene in 2004, added a new nanomaterial to the sensing area [91]. Graphene, with fast electron transportation, high thermal conductivity, excellent mechanical properties and biocompatibility, can avoid the problems associated with metal nanoparticles and CNTs, and also leads to potential applicability in electrochemical biosensors [92]. There are several reviews about the application of graphene in the sensing area [23,92–95], and here the application of graphene for glucose biosensors is reviewed.

### 4.1. Graphene Synthesis

Good graphene based biosensors will depend on how to fabricate high-quality graphene in a reproducible way and even large scale. Three main approaches, so far, have been used to produce graphene.

The first approach is exfoliation of high-quality graphite (HOPG) using pieces of adhesive tape, which eventually leads to some single layers of graphene [91]. This technique produces the best-quality, least-modified forms of graphene, and is the main approach for scientific research. However, to quantify the number of layers and grain size need great patience and it is thus still a major challenge, since the cleaved graphene layers are distributed among carbonaceous fragment containing uncertain numbers of layers.

**Figure 8.**
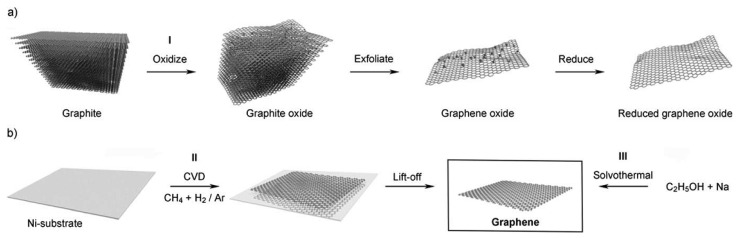
(**a**) The oxidation–exfoliation–reduction process used to generate individual sheets of reduced graphene oxide from graphite; (**b**) schematic representation of the approaches to producing graphene by chemical vapour deposition (Method II, left) or by solvothermal reaction (Method III, right). Reprinted with permission from [23].

Chemical methods have been tried to upscale the graphene yields. As shown in Figure 8(a), the first step is to oxidize graphene under strong acid, which creates a large number of oxygen-containing functional groups, such as carboxyl, epoxide and hydroxyl groups on the graphene surface [96]. These groups make graphene oxide (GO) hydrophilic and are thus able to be dissolved into a single sheet in water or polar organic solvents. The graphene oxide is then reduced by some compounds (like hydrazine) or by heating in a reducing atmosphere to regain the structure and property of graphene. The main disadvantage of this method is the layer produced always contain certain amount of graphene oxide and significant carbon-oxygen bonds [97].

Chemical vapour deposition is the third approach and it can produce large areas of single layer graphene [98] by passing hydrocarbon vapours over metallic substrates, like Ni or Cu, heated to *ca.* 1,000 °C. Kim *et al.* reported that stretchable transparent electrodes can be fabricated through synthesized graphene from the CVD method on Ni substrates. One of the main challenges for the CVD method is in achieving a graphene layer with a monodispersed or controlled number of layers. A Cu substrate is believed, so far, to be the best substrate to make mono-layers of graphene, and Li *et al.* use a self-limiting growth of graphene to obtain mono-layer graphene on centimeter-scale Cu substrates [99].

### 4.2. Graphene Based Enzymatic Sensors

Based on the high electrocatalytic activity of graphene toward H_2_O_2_ and the excellent performance for direct electrochemistry of GOD, graphene could be an excellent candidate for direct electrochemistry of GOD, and an excellent electrode material for glucose based biosensors [95,100].

Shan *et al.* [101] reported the first graphene-based glucose biosensor based upon graphene protected by polyvinylpyrrolidone that could thus be well dispersed in water. It has good electrochemical reduction toward H_2_O_2_. After the GOD is immobilized, the sensor achieved a direct electron transfer between GOD and electrode. A linear glucose response covered from 2 to 14 mM, with good reproducibility (3.2% for 10 successive measurements) and high stability was obtained.

**Figure 9.**
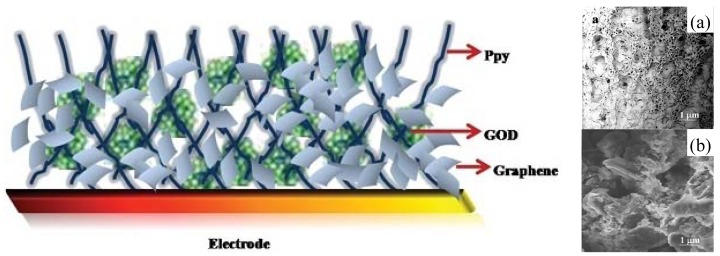
Schematic diagram of Graphene-GOD entrapped within a porous Ppy matrix, the insert (**a**) is the SEM image of porous structure of electropolymerized Ppy; and (**b**) SEM image of Graphene-GOD on the electrode surface. Reprinted with the permission from [102].

Alwarappan *et al.* reported enzyme-doped graphene nanosheets for enhanced glucose biosensing [102]. Graphene nanosheets were chemically synthesized and then covalently conjugated to a GOD. The conjugated graphene/GOD was then immobilized onto the glassy carbon electrode surface already modified with porous polypyrrole (Ppy), as shown in Figure 9. Ppy-graphene-GOD electrodes exhibited an excellent sensitivity of 3 μM, based on the signal/noise = 3. Wu *et al.* [103] developed a novel approach for glucose detection based on the electrocatalytic reduction of oxygen at the GOD-graphene/GC electrode. Upon the addition of glucose, the reduction current decreased. The response displays a linear range from 0.1 to 10 mM with a sensitivity of 110 ± 3 μA·mM^−1^·cm^−2^ and a detection limit of 10 ± 2 μM. Kang *et al.* employed a GOD-Graphene-Chitosan modified electrode [104], where the immobilized enzyme retains its bioactivity and the graphene suspension can be well-dispersed through the help of biocompatible chitosan. A much higher enzyme loading (1.12 × 10^−9^ mol/cm^2^) is obtained as compared to the bare glass carbon surface. The resulting sensors could be used for glucose detection with a high sensitivity (*ca.* 110 ± 3 μA·mM^−1^·cm^−2^), a wide linear range (0.1–10 mM), and a low detection limit (10 ± 2 μM).

### 4.3. Graphene/NP Hybrid Sensor

Another approach for glucose detection is using metal nanoparticles (NP) enhanced graphene electrodes. Wang *et al.* reported the utilization of a graphene-CdS (G-CdS) nanocomposite as a novel immobilization matrix for the enzymes, particularly for GOD [105]. The G-CdS nanocomposite exhibited excellent electron transfer properties for GOD with a rate constant (k_s_) of 5.9 s^−1^. The obtained glucose biosensor displayed satisfactory analytical performance over an acceptable linear range from 2.0 to 16 mM with a detection limit of 0.7 mM, and also limited the effects of interfering species.

Zeng *et al.* [106] reported palladium nanoparticle/chitosan-grafted graphene nanocomposites for construction of a glucose biosensor. Graphene was firstly covalently functionalized with chitosan to improve its biocompatibility and hydrophilicity, and then decorated by palladium nanoparticles (PdNPs) using *in situ* reduction. The sensor exhibited excellent electrocatalytical activity toward H_2_O_2_ and facilitated a high loading of enzymes. A high sensitivity of 31.2 μA·mM^−1^·cm^−2^ for glucose was obtained with a wide linear range from 1.0 μM to 1.0 mM. The low Michaelis-Menten constant (1.2 mM) suggested enhanced enzyme affinity to glucose.

A novel nanocomposite of electrochemically reduced graphene oxide (ERGO) and gold-palladium (1:1) bimetallic nanoparticles (AuPdNPs), without the aid of any reducing reagent was introduced by Yang *et al.* [107]. An ERGO-AuPdNPs nanocomposite showed excellent biocompatibility, enhanced electron transfer kinetics and large electroactive surface area, and was highly sensitive and stable towards oxygen reduction. The resulting sensor displayed a linear range up to 3.5 mM with a sensitivity of 266.6 μA·mM^−1^·cm^−2^.

### 4.4. Graphene Based Non-Enzymatic Sensors

Non-enzymatic sensors can play a new role in the detection of glucose, since they avoid the expensive and fragile enzymes, as we mentioned in the Section 3.6. The development of graphene in the sensing area offers new approaches for non-enzymatic glucose biosensors.

Kong *et al.* developed high-density Au NPs using thionine functionalized graphene oxide as a supporting material via chemically reduction of HAuCl_4_ with sodium citrate [108]. The use of thionine functionalized GO can promote electrostatically adsorbing negatively charged AuCl_4_^−^ on the GO surface and thus a high load of Au NPs can be achieved. GO-thionine-Au nanostructure composites modified glassy carbon electrodes showed remarkably electrocatalytic activity towards the oxidation of glucose, and can thus lead to an enzymeless glucose sensor with a wide linear range between 0.2 to 13.4 mM, and a lower detection limit of 0.05 μM.

Luo *et al.* reported a non-enzymatic glucose sensor by potentiostatically electrodepositing metallic Cu nanoparticles on graphene sheets [109]. The Cu-graphene sheets electrode shows much better electrocatalytic properties for glucose oxidation and detection compared to the unmodified graphene sheets electrode and the Cu/GC electrode. A linear range up to a 4.5 mM glucose level was achieved by this Cu-graphene electrode sensor, with a detection limit of 0.5 μM (signal/noise = 3) at a detection potential of 500 mV.

Xiao *et al.* introduced a non-enzymatic glucose sensor through a one-step electrochemical synthesis of PtNi nanoparticle-graphene nanocomposites [110]. The nanocomposites exhibit several unique features including well-dispersed NPs with alloy features, high NPs loading, and effective reduction of graphene oxide. Under the physiological condition, a linear range up to 35 mM with a sensitivity of 20.42 μA·cm^−2^·mM^−1^ at a substantially negative potential (*i.e.*, −0.35 V) was obtained. One of the great efforts to operate under this negative potential eliminates the impact from the oxidation of common interfering species.

## 5. Conclusions and Future Directions

The worldwide increase in the number of diabetic patients has encouraged scientists to put great efforts into the field of glucose biosensors. So far, there are three generations in the development of glucose biosensors, and the market leaders are still based on second generation sensor technology using mediator modified GOD sensors. Carbon nanomaterials have similar dimensions as redox proteins, and can be used as effective electrical wiring/connectors with redox enzymes. This is still a second generation technique, and is one of the most promising directions for enzymatic glucose biosensors. For biosensing applications, CNTs and graphene demonstrate faster response times and higher sensitivity than traditional electrodes at extremely low working potentials. However, better control of the chemical and physical properties of carbon material based biosensors is still needed. For example, the separation process for different type of CNTs, the miniaturization of the sensor, the possibility of toxicity, *in vivo* stability *etc.* still needs to be addressed to meet future requirements. In order to address some of the above issues, ideas on the future direction about CNT-based biosensors have been summarized.

Cost-effective, large scale fabrication of CNT nanoelectrode arrays (NEAs) is one attractive direction [111–113]. Such arrays of nanoelectrodes are able to produce much higher currents than a single nanoelectrode. This would avoid the need for expensive electronic devices and thus improve the signal to noise ratio, leading to ultrasensitive electrochemical sensors for chemical and biological sensing. Tu *et al.* reported a low-site density carbon nanotube based nanoelectrode array, and epoxy resin was spin-coated to create the electrode passivation layer effectively reducing the electrode capacitance and current leakage [112]. Further improvement in these promising CNT-NEAs for biosensor applications requires tuneable and predictable assembly with well-ordered structures. Novel nano-technology such as soft lithography, nanoimprint lithography and highly ordered porous anodising alumina template are able to help achieve these.

Another promising approach for the direct detection of biological species is field-effect transistors which offer several possible advantages, like forming a semi-conducting channel using SWCNTs; absorption of molecules on the FET surface and manipulation at the molecular level with cells. Single cell analysis has become a highly attractive tool for investigating cellular contents. Villamizaar *et al.* revealed a fast, sensitive and label-free biosensor based on a network of SWCNTs which act as the semi-conducting channel for the selective determination of salmonella Infantis [114]. Since the semiconductor properties of SWCNTs are critical to form FETs for biosensing, a continuing problem is that the sample produced by all CNT synthesis methods contains both semiconducting and metallic nanotubes. Recent reports addressing the separation and purification of semiconducting and metallic CNTs are undoubtedly a landmark advance, but higher yield and purity is still desired [115–117].

A very important issue related to the integration of CNTs into biological cells and tissues is the need to study their cytotoxicity towards biological species. Contradictory results have been reported in recent research on the toxicity of CNTs. Poland *et al.* demonstrated that asbestos-like pathogenic behaviour was associated with CNTs. They showed that the toxicity depends on length and thus suggested the use of commercially long CNTs [118]. Pantarotto *et al.* have shown that SWCNTs will be toxic to mammalian cells beyond 10 μmol/L (*ca.* 0.022 mg/mL) [119]. Meanwhile, Kam *et al.* reported that SWCNTs are nontoxic up to a high concentration, 0.05 mg/mL [120]. Therefore, an in-depth systematic and long-term study of the effect of CNTs on human cells and tissues as well as information related to safety issues still needs to be carried out. Much work is still required in this field.

Regardless of the contradictory results on toxicity of CNTs as discussed above, development of CNTs in non-particulate forms such as continuous CNT fibers is a safe way to avoid the potential risk of CNT leaching, especially when used in implantable electrodes for *in vivo* testing. Although CNT fibers have high strength (1.8–3.0 GPa), stiffness (330 GPa) and good electrical conductivity (8.3 × 10^5^ s·m^−1^), the development of CNT fibers into the biosening area is still at early stage. For example, highly porous network structures of fibers and unique brush-like nanostructure fiber ends lead to short response times in amperometric test and fast electron transfer between the redox center of the enzyme and CNT fiber [68]. CNT fibers open for the way for the safe use of CNTs in the biosensing area when dealing with cells and tissue, especially for implantable biosensors. However, it is fair to say that there is still a long way to the full utilization of CNT fibers for biosensing applications.

Regarding two dimensional graphene based biosensors and devices; they have exhibited good sensitivity and selectivity towards the detection of glucose. How to make consistent and reproducible graphene and sensors in large volume, based on such material, is still a great concern. The first challenge is to develop well-controlled synthesis and processing of graphene. As mentioned above, graphene isolated from HOPG by pieces of adhesive tape has proven to be the best quality material so far. However, controlling the number of layers, minimizing folding and bending during processing, and limiting substrate effects are all factors that need to be addressed [23]. High surface area graphene prepared by CVD process is one of the most compatible to the MEMS/CMOS process, and we believe it will play key role in later device fabrication. Another challenge to make quality devices is avoiding surface contamination. Graphene is highly hydrophobic and thus is easily contaminated by various species, particularly hydrocarbons [121,122]. The two dimensional structure exacerbates the contamination issue due to the large lateral surface area. For example, the photoresists and solvents used in the microfabrication process to construct devices are difficult to remove. Undesired contamination should be avoided during processing to eliminate its effect on the sensing response.

Non-enzymatic sensors show enhanced sensitivity and detection limits, while avoiding the expensive and fragile enzymes in the system, and thus they are a promising choice for the third generation glucose sensor. Over the past decade, the surge in interest and demand of using nanomaterials in non-enzymatic glucose sensors has increased and the approaches include the use of carbon nanomaterials: CNTs and graphene. Nanoporous and microporous metals or alloys, for example, PtPb nanoporous electrode [83] and nanoporous Au [86] can be used as the non-enzymatic electrode itself. The major parameter for the electrode is the roughness factor, the greater the surface roughness, the greater the electrochemical activity [123]. For carbon nanomaterials, it is critical to combine with metal nanoparticles to construct a hybrid sensor, where nickel and copper are commonly used. The great performance of the CNT/metal nanocomposite electrodes toward the oxidation of glucose is mainly attributed to the increase of the electroactive working electrode surface by the use of carbon nanomaterials and the electrocatalytic activity by the homogeneous dispersion of the metal nanoparticles. One of the great concerns for a non-enzymatic sensor is the CNT/metal nanocomposite electrode has to be used in a base environment, where an OH- group is strongly needed to form higher oxides, such as Ni/CuOOH. Therefore, they are not ready yet to replace traditional blood glucose sensors in either pH neutral or acidic conditions.

## Figures and Tables

**Table 1.** Comparison of analytical performance of CNT/Ni nanocomposite sensor with different nonenzymatic glucose biosensors [79].

**Electrode type**	**Applied potential** **(mV)**	**Sensitivity** **(μA·mM^−1^·cm^−2^)**	**Linear range**	**Detection Limit**	**Reference**
Nanoporous PtPb	−80	10.8	1–16 mM	N/A	[83]
Mesoporous Pt	+400	9.6	0–10	N/A	[84]
Pt-Pb/CNTs	+300	17.8	Up to 11 mM	1.0 μM	[85]
Porous Au	+350	11.8	2–10 mM	5 μM	[86]
MnO_2_/MWCNTs	+300	33.19	10 μM–28 mM	10 μM	[75]
Cu/MWCNTs	+650	251.4	0.7–3.5 mM	0.21 μM	[73]
Cu nanocubes/MWCNTs	+550	1,096	1 μM–7.5 mM	1 μM	[87]
NiO/MWCNTs	+500	1,770	10 μM–7 mM	2 μM	[81]
NiNP/SMWNTs	+400	1,438	1 μM–1 mM	0.5 μM	[88]
Ni(OH)_2_/CILE	+550	202	0.05–23 mM	6 μM	[89]
Electrospun NiCFP	+600 V	420.4	2 μM–2.5 mM	1 μM	[90]
Ni nanowire arrays	+550 V	1,043	0.5 μM–7 mM	0.1 μM	[76]
CNT/Ni	+525	1,433	5 μM–7 mM	1 μM	[79]
